# Comparing the effect of laparoscopic and robotic stapling on clinical outcomes, efficiency, and costs of robot-assisted Roux-en-Y gastric bypass

**DOI:** 10.1007/s11701-025-02223-6

**Published:** 2025-02-08

**Authors:** Juliane O. Hafermann, Jarrod D. Phelps, Mahmoud F. El Tayash

**Affiliations:** 1 Coreva Scientific GmbH & Co. KG, Königswinter, Germany; 2 Grinnell Regional Medical Center, UnityPoint Health, 210 4th Ave, Grinnell, IA 50112 USA

**Keywords:** Roux-en-Y gastric bypass, Robot-assisted surgery, Surgical stapling, Laparoscopic stapler, Robotic stapler

## Abstract

Roux-en-Y gastric bypass (RYGB) is a commonly used surgery to successfully and durably treat obesity that is increasingly performed robotically. The anastomoses created during robot-assisted RYGB are usually stapled, either using laparoscopic or robotic staplers. This study aimed to compare the peri-operative efficiency, costs, and clinical outcomes of laparoscopic and robotic staplers in robot-assisted RYGB. This retrospective study analyzed patients who underwent robot-assisted RYGB (with or without hiatal hernia repair) with the Da Vinci Xi robotic system in a private practice in the United States. The gastric pouch and anastomoses were created either with robotic SureForm™ staplers or laparoscopic Endo GIA™ staplers; enterotomies and incisions were closed with sutures. The primary outcome was procedure time. The secondary outcomes were instrument exchanges and reloads used, stapling costs, length of stay, and complications within 3 months. Of the 105 patients included, 50 patients were treated with robotic staplers and 55 with laparoscopic staplers. None were lost to follow-up. Laparoscopic staplers were more efficient than robotic ones: they significantly reduced procedure times across all analyzed categories as well as the number of instrument exchanges and reloads needed per surgery. There was no difference in the length of hospital stay, and no complications were reported. The higher number of reloads and the higher unit cost resulted in higher total stapling costs for robotic compared to laparoscopic staplers. While robotic staplers in robot-assisted RYGB are safe and feasible, laparoscopic staplers improve efficiency, reduce resource use, and lower costs without compromising patient safety.

## Introduction

The number of people living with obesity worldwide has more than doubled between 1990 and 2022, and more than 1 billion people were living with obesity in 2022 [1]. The most effective treatment for obesity is metabolic bariatric surgery (MBS) [2]. Roux-en-Y gastric bypass (RYGB) is the second most commonly performed MBS procedure, accounting for 29.5% of all MBS procedures and nearly half of all revisional MBS procedures worldwide [3]. Most MBS procedures are performed as laparoscopic or robot-assisted surgery [3]. In the USA, approximately 30% of MBS procedures are performed robotically [3].

Most studies agree that laparoscopic and robot-assisted MBS are equally effective [4–9]. Some studies suggest that robot-assisted RYGB may improve certain outcomes such as a shorter length of stay [9], fewer anastomotic leaks [5], less post-operative bleeding, and fewer blood transfusions [10]. However, the robot-assisted approach may also lead to longer operative times and higher costs [5, 7].

In both laparoscopic and robot-assisted RYGB surgeries, anastomoses are stapled [11, 12]. During robot-assisted surgery, stapling can be done by the robot using robotic staplers or a human surgical assistant or surgeon using laparoscopic staplers. Studies comparing these two stapler types so far have come to different conclusions regarding their efficacy and safety: both stapler types seem to perform equally well in rectal cancer surgery [13], whereas robotic staplers may provide an advantage in robotic lobectomy [14]. Although robotic stapling in RYGB was found to be safe and feasible, robotic staplers were associated with a longer operative time, more stapler firings, and higher stapling costs than laparoscopic staplers [15]. That study compared robotic SureForm™ staplers to laparoscopic staplers by Ethicon for the creation of the gastric pouch [15]. In contrast, the present study aims to compare the peri-operative efficiency, stapling costs, and clinical outcomes of robotic SureForm™ staplers by Intuitive Surgical to laparoscopic Endo GIA™ staplers by Medtronic for the creation of both gastric pouch and anastomoses during RYGB.

## Materials and methods

### Study design

This retrospective study analyzed the records of patients who underwent MBS between May 2022 and November 2023 at the private Grinnell Regional Medical Center in Iowa, USA. Patients were included if they underwent primary robot-assisted RYGB both with and without hiatal hernia repair. In each patient, the creation of the gastric pouch and the anastomoses was performed with either robotic staplers or laparoscopic staplers. The surgical team avoided difficult cases with a BMI > 50 kg/m^2^ during the first ten cases with the laparoscopic stapler until they had become more familiar with the hybrid approach (i.e., using a laparoscopic stapler during robot-assisted surgery) and felt confident performing a more technically challenging procedure. No further exclusion criteria were applied after this initial learning curve with the laparoscopic stapler. As this was a retrospective observational study of anonymous data without any patient identifiers or other identifying information, no ethical approval is required according to 45 CFR 46 §46.104(d)(4)(ii). The study was performed in accordance with the Declaration of Helsinki [16].

### Surgical technique

For all procedures, the patient was placed in a supine position. Pneumoperitoneum was established using a Veress needle at Palmer’s point. Six ports were installed in all cases of both study arms: four 8.5 mm robotic ports, one port for the camera, and one 12 mm port used by either the robotic stapler or the bedside assistant. A liver retractor was placed through an epigastric incision to expose the hiatus and fundus of the stomach. The stomach was decompressed with a Gastric Balloon Suction Catheter (Reshape Lifesciences Inc.) with a size between 29 and 46 Fr and filled with 20 ml fluid, and the Da Vinci Xi robotic system (Intuitive Surgical) was docked. Hiatal hernia repair was performed if indicated, using a 360° dissection in all cases and ensuring a minimum of 2–3 cm of intra-abdominal esophagus length. The neurovascular bundle along the inner gastric curve was robotically dissected just below the level of the balloon. The gastric pouch and anastomoses were created using either robotic SureForm™ staplers (Intuitive Surgical), or the laparoscopic Signia™ stapling system and Endo GIA™ reloads with TriStaple™ technology (Medtronic). The gastric pouch was created either by the robot using one 60 mm green and two to three 60 mm blue robotic staplers or by the bedside assistant using one 45 mm purple and two 60 mm tan laparoscopic staplers. The gastrojejunal and jejunojejunal anastomoses were created using 60 mm white robotic staplers or 60 mm tan laparoscopic staplers to create a biliopancreatic limb of approximately 100 cm and an alimentary limb of approximately 150 cm.

The enterotomies were closed in two layers: the enterotomy of the gastrojejunal anastomosis was closed using an interrupted braided nylon suture followed by a running 2–0 polydioxanone suture; the enterotomy of the jejunojejunal anastomosis was closed by a running 2–0 vicryl suture and a running 2–0 polydioxanone suture. The mesenteric windows were closed with running 2–0 silk sutures. An intra-operative gastric air pressure leak test was performed and, if no leaks were detected, the port sites were closed with a 1–0 vicryl suture using a fascia closure device. The skin was approximated with a 4–0 monofilament absorbable suture and sealed with skin glue.

All surgeries were performed by a highly experienced surgeon who previously performed more than 700 cases of laparoscopic and robot-assisted RYGBs, supported by a highly experienced certified physician assistant with 14 years of experience providing bedside assistance in both laparoscopic and robot-assisted RYGBs.

### Stapling procedure

The surgeons switched from using robotic staplers to using laparoscopic staplers in February 2023, initially due to supply-chain issues for the robotic staplers during the COVID-19 pandemic, but later due to surgeon preference. For robotic stapling, the bedside assistant attached the robotic stapler to the robot arm which was navigated to the stapling site by the surgeon using the robot. The stapler was clamped on the targeted tissue and fired. An automatic delay of approximately 20 s occurs when the stapler is triggered during which the robotic stapler calculates tissue thickness and compression before actually firing the stapler. For laparoscopic stapling, the stapler was positioned by the bedside assistant guided by the surgeon at the console. The device was clamped and the appropriate tissue compression was confirmed on the stapler display before the stapler was fired. Unlike the robotic stapler, there is no automatic delay in firing the laparoscopic stapler. All staple lines were checked for bleeding at the end of the procedure.

### Outcomes

The primary outcomes were procedure times with the following definitions: The operating room (OR) time was the time the patient was in the OR. The console time was the time the surgeon spent at the robot console. It can further be split into the active time (the time the surgeon operated the robot), and the waiting time (the time the robot arms were not active, e.g. to replace instruments). All times were taken from the My Intuitive app (Intuitive Surgical) containing the surgical robot logs, which counts times when the robot was performing calculations and compressing the tissue without firing the stapler not as waiting time but active time.

The secondary outcomes were the number of exchanges of the instrument attached to a robotic arm per surgery, number of reloads used, total stapling costs (calculated from the costs per reload and stapler handle and the number of reloads used), length of stay, and complications within three months of follow-up.

### Statistical analysis

Continuous variables were expressed as mean ± standard deviation or median and interquartile range (IQR). Differences between the two groups were the mean difference ± pooled standard error. Categorical variables were expressed as counts and percentages. The statistical significance of pairwise comparisons was determined using *t*-test or Chi-squared test. Subgroup analysis was performed for patients with and without hiatal hernia repair.

## Results

### Study population

A total of 105 patients underwent robot-assisted RYGB; 50 patients (47.6%) were treated with robotic staplers and 55 patients (52.4%) with laparoscopic staplers. The demographics of both groups were similar regarding age, sex, weight, BMI, and hiatal hernia repair (Table 1).

**Table 1:** Baseline characteristics of the study population

Characteristics	Robotic stapler (*n* = 50)	Laparoscopic stapler (*n* = 55)	Difference (mean ± SEM)	*p*-value
Age, years (mean ± SD)	44.78 ± 10.53	42.69 ± 11.21	2.09 ± 2.13	0.3287
Sex (*n*, %)				
Male	8 (16.0%)	4 (7.3%)		0.1604
Female	42 (84.0%)	51 (92.7%)		
Hiatal hernia repair (*n*, %)				
Yes	20 (40.0%)	22 (40.0%)		1.0000
No	30 (60.0%)	33 (60.0%)		
Weight at baseline, lbs. (mean ± SD)	288.84 ± 72.58	290.75 ± 66.87	−1.91 ± 13.61	0.8889
BMI at baseline, kg/m^2^ (mean ± SD)	46.70 ± 9.83	47.07 ± 8.25	−0.37 ± 1.77	0.8332

*BMI* Body Mass Index, *SD* Standard deviation, *SEM* Standard error of the mean

### Procedure efficiency and clinical outcomes

The use of laparoscopic staplers significantly increased surgical efficacy by reducing procedure time and instrument exchanges (Table 2). It significantly shortened OR time from 124.24 ± 26.98 min to 106.62 ± 28.97 min (*p* = 0.0017), console time from 93.98 ± 24.16 min to 77.75 ± 26.52 min (*p* = 0.0015), active time from 83.68 ± 21.90 min to 69.80 ± 23.24 min (*p* = 0.0022), and waiting time from 10.30 ± 4.90 min to 7.95 ± 6.23 min (*p* = 0.0348). The time savings are likely due to laparoscopic staplers needing only a median 7 (IQR 5–8) instrument exchanges compared to the median 18 instrument exchanges (IQR 17–21) required with the robotic staplers (*p* < 0.0001), and using a median 6 (IQR 6–7) laparoscopic stapler reloads per procedure compared to a median 7 (IQR 6–7) robotic stapler reloads (*p* = 0.0002). The length of stay was comparable between groups (robotic stapler: 1.20 ± 0.45 days, laparoscopic stapler: 1.11 ± 0.31 days, *p* = 0.2308). None of the patients showed any intra- or post-operative complications within 3 months.

**Table 2:** Surgical efficiency and costs associated with robotic and laparoscopic staplers in robot-assisted RYGB

Outcome	Robotic stapler (*n* = 50)	Laparoscopic stapler (*n* = 55)	Difference (mean ± SEM)	*p* value
Total OR time, min (mean ± SD)	124.24 ± 26.98	106.62 ± 28.97	17.62 ± 5.48	0.0017
Console time, min (mean ± SD)	93.98 ± 24.16	77.75 ± 26.52	16.23 ± 4.97	0.0015
Active time, min (mean ± SD)	83.68 ± 21.90	69.80 ± 23.24	13.88 ± 4.42	0.0022
Waiting time, min (mean ± SD)	10.30 ± 4.90	7.95 ± 6.23	2.35 ± 1.10	0.0348
Instrument exchanges, n (median, IQR)	18 (17–21)	7 (5–8)	12.32 ± 0.56	< 0.0001
Reloads used, n (median, IRQ)	7 (6–7)	6 (6–7)	0.41 ± 0.11	0.0002
Length of stay, days (mean ± SD)	1.20 ± 0.45	1.11 ± 0.31	0.09 ± 0.08	0.2308
Intra-operative complications, n (median, IQR)	0 (0–0)	0 (0–0)	0	–/–
Post-operative complications at 3 months, n	0 (0–0)	0 (0–0)	0	–/–
Total stapling costs, USD (mean ± SD)	2174.55 ± 148.39	1476.60 ± 89.10	697.95 ± 23.64	< 0.0001

*BMI* Body Mass Index, *IQR* Interquartile range, *SD* Standard deviation, *SEM* Standard error of the mean, *USD* United States Dollars

### Costs

The unit cost of the robotic stapler handle was USD 451 more expensive than the laparoscopic stapler handle. In addition, the unit cost of the robotic stapler reload was between USD 20 and USD 94 higher than those of the laparoscopic reloads (depending on size). Using the robotic stapler required significantly more reloads per patient (median 7, IQR 6–7) than the laparoscopic stapler (median 6, IQR 6–7, *p* = 0.0002) (Table 2). The higher unit costs and the higher number of reloads per patient resulted in significantly higher total stapling costs for robotic staplers (USD 2,175 ± 148) than for laparoscopic staplers (USD 1,477 ± 89, *p* < 0.0001).

### Subgroup analysis: hiatal hernia repair

Hiatal hernia repair was performed in 40.0% of patients in both the robotic stapler group (*n* = 20) and the laparoscopic stapler group (*n* = 22) (*p* = 1.0). Laparoscopic staplers significantly reduced the procedure times in both the overall population (from 124.24 ± 26.98 min to 106.62 ± 28.97 min, *p* = 0.0017) and the subgroup without simultaneous hiatal hernia repair (from 116.87 ± 28.42 to 96.94 ± 25.65, *p* = 0.0051) (Table 3). However, repairing the hiatal hernia took longer in the laparoscopic than in the robotic stapler group, so that the time savings with the laparoscopic stapler were no longer statistically significant.

**Table 3:** Subgroup analysis comparing outcomes between patients with and without simultaneous hiatal hernia repair in the robotic and laparoscopic stapler groups

Outcome	Robotic stapler				Laparoscopic stapler				*p* value laparoscopic vs robotic stapler	
Hiatal hernia repair?	Yes (*n* = 20)	No (*n* = 30)	Difference	*p*-value	Yes (*n* = 22)	No (*n* = 33)	Difference	*p*-value	Yes	No
Total OR time, min (mean ± SD)	135.30 ± 20.73	116.87 ± 28.42	18.43	0.0163	121.14 ± 28.07	96.94 ± 25.65	24.20	0.0017	0.0689	0.0051
Console time, min (mean ± SD)	105.65 ± 18.80	86.20 ± 24.45	19.45	0.0042	93.73 ± 27.34	67.09 ± 20.09	26.64	0.0001	0.1055	0.0014
Active time, min (mean ± SD)	93.95 ± 18.90	76.83 ± 21.33	17.12	0.0055	83.50 ± 24.04	60.67 ± 17.81	22.83	0.0002	0.1237	0.0020
Waiting time, min (mean ± SD)	11.70 ± 4.46	9.37 ± 5.02	2.33	0.0992	10.23 ± 7.44	6.42 ± 4.80	3.80	0.0250	0.4374	0.0209
Instrument exchanges, *n* ( mean ± SD)	20.05 ± 2.84	18.43 ± 3.61	1.62	0.0985	8.00 ± 2.64	5.94 ± 1.58	2.06	0.0006	< 0.0001	< 0.0001
Reloads used, *n* (mean ± SD)	6.60 ± 0.60	6.77 ± 0.63	−0.17	0.3527	6.36 ± 0.49	6.24 ± 0.44	0.12	0.3414	0.1728	0.0004
Length of stay, days (mean ± SD)	1.20 ± 0.41	1.20 ± 0.48	0.00	1.0000	1.23 ± 0.43	1.03 ± 0.17	0.20	0.0215	0.8343	0.0778
Total stapling costs, USD (mean ± SD)	2150.40 ± 144.48	2190.65 ± 151.19	−40.25	0.3527	1488.72 ± 93.83	1468.52 ± 86.32	20.19	0.4153	< 0.0001	< 0.0001

*OR* Operating room, *SD* Standard deviation

## Discussion

This study demonstrated that although the use of robotic staplers is safe and feasible in robot-assisted RYGB, it requires more stapler firings and more frequent instrument changes, leading to longer operative times. Due to their higher resource use and higher unit costs, robotic staplers also significantly increased stapling costs.

The results of this study are similar to a previous study comparing laparoscopic and robotic staplers in robot-assisted RYGB, which also reported longer operative times and higher stapling costs [15]. That study attributed the longer operative time to a relatively high rate of unsuccessful clamping attempts (19%) with the robotic stapler [15]. Our study did not record the number of failed clamping attempts, although that could certainly have contributed to the increase in operating time we observed. Additionally, we identified the higher number of instrument exchanges and the additional stapler firings needed with the robotic stapler as responsible for the longer operative time. The increase in instrument exchanges in the robotic stapler group is due to having to attach the stapler to the robotic arm and reloading it between firings, as well as having to switch to other instruments (e.g., for grasping, dissection, or suturing) that may be needed between and after the different stapling steps.

A few studies comparing laparoscopic to robotic staplers in other types of surgery have been published [17], and they came to varying conclusions. No clear benefit of the robotic stapler has been observed in colorectal surgery [13]. Still, surgeons may value the additional maneuverability offered by the robotic stapler if they are working in confined spaces like the pelvis, which is more difficult to reach than the relatively easy access during RYGB. In thoracic surgery, one study found no difference between robotic and laparoscopic staplers in lung resection [18], whereas another reported a lower risk of bleeding and conversion to open surgery after lobectomy with robotic staplers [14]. The effect of robotic instead of laparoscopic staplers on operative time across these studies is inconclusive, with some studies reporting significantly longer operative times [15, 18–20] whereas others found no significant difference [14, 21–23].

Hiatal hernia repair increased procedure time. This increase was larger in the laparoscopic stapler group, possibly due to larger or more complicated hernias in this patient population. Therefore, the time savings with laparoscopic staplers were, though numerically still observed, no longer statistically significant. The reason behind this is likely twofold: the time difference between the laparoscopic and robotic stapler groups was reduced and the smaller sizes of the patient subgroups may no longer be powered to detect a significant difference.

Similar to our findings, some studies reported higher material costs [15] or higher procedure and total hospitalization costs [19] for robotic staplers. However, other studies reported comparable total and material costs [14] or even lower material costs for robotic staplers [23]. In that particular study, the lower material costs resulted from smaller laparoscopic staplers and fewer stapler firings per patient in the robotic group [23], whereas larger laparoscopic staplers were used and more robotic stapler firings were needed in our study. Thus, the higher costs of robotic staplers observed in this study are driven by both the inherently higher prices of robotic staplers and reloads, as well as the higher number of reloads required.

The evaluation of stapler costs was limited to the direct costs of the stapling device and the stapler reloads. The cost calculation did not include other materials such as trocars, trocar reducers, and/or stapler sheaths. It furthermore did not consider the additional indirect costs incurred by the longer operative time observed in the robotic stapler group, nor the costs associated with the surgical assistant operating the laparoscopic stapler. For a better understanding of all associated costs in context and not just the direct stapler material costs, a health economic analysis would be needed.

Further limitations of our study are the retrospective nature, leading to a potential risk of bias and a low level of evidence. Furthermore, the number of clamping attempts was not recorded, which could have contributed to the difference in operative time. As the surgeons switched from using robotic to laparoscopic staplers after 50 patients, the learning curve for the use of laparoscopic staplers might have affected the outcomes of that group. The study was too small and follow-up too short to be able to detect differences in intra- or post-operative complications. Although the relatively small population size was sufficient to detect the difference in operative time between the groups, it left subgroup analyses for hiatal hernia repair potentially underpowered. To determine if and how other parts of the surgery that do not use stapling (like hiatal hernia repair in this case) influence the time saving effect of laparoscopic staplers in RYGB, a larger study that controls for hernia size and complexity and that is sufficiently powered to detect a difference between the subgroups would be needed. Finally, these surgeries were performed by a single surgeon and surgical assistant and may depend on other factors that cannot be generalized to all settings.

## Conclusion

This retrospective study showed that although using robotic staplers in robot-assisted RYGB is feasible and safe, the use of laparoscopic staplers streamlined the procedure. In our experience, laparoscopic staplers were more efficient and associated with less resource use and lower stapling costs than robotic staplers without affecting patient safety. Further research is needed to determine to which extent this reported experience is generalizable to other surgical teams and settings.

## Data Availability

The data that support the findings of this study are not openly available due to reasons of sensitivity and are available from the corresponding author upon reasonable request. Data are located in controlled access data storage at Grinnell Regional Medical Center.
